# Microstructure observation and flavor substances excavation of Yunyan 87 tobacco leaves with different oil contents

**DOI:** 10.3389/fpls.2025.1537924

**Published:** 2025-03-05

**Authors:** Xianfeng Hu, Wei Xu, Yubo Zhang, Shouhui Pan, Yanlan Xie, Rui Liao, Shenggang Yang, Youxiang Wu, Daomao Deng

**Affiliations:** ^1^ College of Agriculture, Anshun University, Anshun, Guizhou, China; ^2^ Raw Material Supply Center, China Tobacco Guizhou Industrial Co Ltd., Guiyang, Guizhou, China; ^3^ Production Technology Center, Guizhou Province Tobacco Company Anshun Company, Anshun, Guizhou, China

**Keywords:** flavor substances, hexadecanoic acid methyl ester, nonanoic acid methyl ester, pseudoionone, tobacco oil content

## Abstract

**Introduction:**

The oil content of tobacco leaves is intimately associated with their aromatic characteristics. This study aims to explore the microstructure and distinctive flavor substances of Yunyan 87 high-oil-content tobacco leaves.

**Methods:**

The microstructure and characteristic flavor substances of Yunyan 87 tobacco leaves with different oil contents were analyzed using scanning electron microscope (SEM) and comprehensive two-dimensional gas chromatography coupled with time-of-flight mass spectrometry (GC×GC-TOF MS).

**Results:**

The results indicate that the surface of high-oil tobacco leaves was characterized by a high density of glandular hairs, primarily composed of short-stalked glandular hairs featuring enlarged glandular heads. A total of 1551 flavor substances were detected in high-oil tobacco leaves, compared to 1500 metabolites were identified in low-oil tobacco leaves. Among these flavor substances, eight exhibited up-regulated, while three were down-regulated. Notably, the oil-related substances hexadecanoic acid methyl ester and the aroma-related substances nonanoic acid methyl ester and pseudoionone were found to be significantly more abundant in high-oil tobacco leaves compared to their low-oil counterparts. Consequently, hexadecanoic acid methyl ester may serve as a reliable indicator for evaluating the oil content in tobacco leaves, while nonanoic acid methyl ester and pseudoionone could play crucial roles as flavor substances influencing the aroma of tobacco leaves.

**Discussion:**

These findings provide a theoretical foundation for future research on the regulatory mechanisms underlying the synthesis of aroma-producing flavor substances in tobacco leaves.

## 1 Introduction

Tobacco (*Nicotiana tabacum* L. ) serves as a cash crop that plays a crucial role in the economies of numerous countries (Wang and Yan, 2013; Yin et al., 2019). The aroma of tobacco represents a complex perception by the human olfactory system in response to the volatile organic compounds (VOCs) emitted by combusted tobacco leaves. High-quality tobacco leaves are characterized by a mellow taste, distinctive flavor, and rich aroma. The precursors of tobacco aroma include sugars, pigments, amino acids, polyphenols, and alkaloids, all of which make a significant contribution to the aroma quality of tobacco products (Zhang et al., 2013; Qin et al., 2020; Liu et al., 2022). The flavor substances in tobacco leaves can be categorized into alcohols, aldehydes, ketones, acids, esters, and phenols based on their functional groups (Forehand et al., 2000). Researchers employed non-targeted metabolomics to identify the substances responsible for aroma generation before and after the baking of K326 tobacco and to assess their relevance to the quality of tobacco leaves. Among the 584 metabolites analyzed, 44 were identified as aroma - related metabolites, including alcohols, aldehydes, phenols, and organic acids (Zou et al., 2023). The variety, maturity, cultivation conditions, altitude, climate, and processing technology of tobacco significantly influence the composition and concentration of its aromatic components. Yan et al (Yan et al., 2020). reported that the application of active organic carbon in the soil enriches the aromatic substances present in tobacco leaves. Compared with pure chemical treatment, the combined application of biochar and chemical fertilizers as well as high - carbon and chemical fertilizer treatment can increase soil carbon and nitrogen contents, and enhance the levels of neutral aromatic substances in tobacco leaves by 19. 38% and 28. 90% respectively (Yan et al., 2020a). The researchers discovered that the abundance of aromatic substances in Changchun tobacco leaves sourced from Jilin Province was greater than that in Nanxiong tobacco leaves obtained from Guangdong Province (Qin et al., 2021). Furthermore, the aroma of tobacco leaves is closely associated with their oil content, leaves with higher oil content exhibit a more pronounced aroma. An enhanced oil content not only augments the softness of tobacco leaves but also elevates their grade and quality, thereby yielding a more robust flavor. Nevertheless, studies on the differences in the microstructures and flavor substances among tobacco leaves with diverse oil contents are rarely reported.

Flavor omics is widely used to analyze the flavor characteristics of various foods, including alcohol, tea, fruits, chili peppers, and meat products, etc (Huang et al., 2022; Wang et al., 2022; Yang et al., 2022; Gu et al., 2023; Ruan et al., 2023; Li et al., 2024; Lin et al., 2024). Comprehensive two-dimensional gas chromatography time-of-flight mass spectrometry (GC×GC-TOF MS) is characterized by high throughput, precision, sensitivity, and reproducibility, and is thus widely applied in flavor omics. Chen et al (Chen et al., 2024). conducted a comprehensive analysis of the flavor characteristics of rapeseed protein isolates treated with phytase and ethanol using GC×GC-TOF MS. They found that this treatment led to a decrease in carboxylic acid substances with acidic odors and an increase in ester substances that contribute to the fruity and sweet aroma of the rapeseed protein isolates. Schwanz et al (Schwanz et al., 2019). employed GC×GC-TOF MS in combination with chemometrics to analyze the chemical sensory markers in cigarette smoke derived from diverse tobacco varieties, thereby establishing a correlation among key chemical sensory markers, tobacco varieties, and cultivation practices. In conclusion, GC×GC-TOF MS characterized by prominent advantages including high - resolution capability, high sensitivity, and the capacity to rapidly analyze numerous compounds in complex samples, provides a comprehensive analytical methodology for the investigation of flavor substances.

The oil content of tobacco leaves is a crucial indicator for quality evaluation. Tobacco leaves with higher oil content are characterized by improved aroma quality. This paper is aimed at exploring the microstructure and distinctive flavor substances of Yunyan 87 high-oil-content tobacco leaves. The Yunyan 87 tobacco leaves, characterized by different oil contents, were analyzed using scanning electron microscope (SEM) and GC×GC-TOF MS to elucidate the microstructure and distinctive flavor substances present in high-oil content Yunyan 87 tobacco leaves. This research provides a theoretical foundation for further exploration of the synthesis and regulatory mechanisms underlying aroma-producing characteristic flavor substances in tobacco leaves.

## 2 Materials and methods

### 2.1 Materials

Ethanol (99. 8%) was procured from Aladdin Holding Group Co. , Ltd. (Beijing, China). Sodium chloride, anhydrous ethanol, and isoamyl acetate were sourced from Sinopharm Chemical Reagents Co. , Ltd. (Shanghai, China). Electron microscope stationary liquid and phosphate buffered saline (PBS) were obtained from Wuhan Xavier Biotechnology Co. , Ltd. (Wuhan, China), while osmic acid was purchased from Shanghai Kanglang Biotechnology Co. , Ltd. (Shanghai, China).

### 2.2 Sensory evaluation of tobacco leaves

Five tobacco sensory evaluation experts from Guizhou Zhongyan Industrial Co. , Ltd. evaluated ten Yunyan 87 dry tobacco leaf samples for indicators such as aroma quality, aroma quantity, miscellaneous gas, thrill, sweet feeling, and oil content. The specific criteria are as follows:

1. *Aroma quality*: relatively good (7. 6–9. 0), above-average (6. 1–7. 5), moderate (4. 6–6. 0), below-average (3. 1–4. 5), poor (≤3).
2. *Aroma quantity*: sufficient (7. 6–9. 0), adequate (6. 1–7. 5), available (4. 6–6. 0), less (3. 1–4. 5), very less (≤3).
3. *Miscellaneous gas*: very light (7. 6–9. 0), light (6. 1–7. 5), moderate (4. 6–6. 0), heavy (3. 1–4. 5), very heavy (≤3).
4. *Thrill*: very small (7. 6–9. 0), small (6. 1–7. 5), moderate (4. 6–6. 0), large (3. 1–4. 5), very large (≤3).
5. *Agreeable aftertaste*: comfortable (7. 6–9. 0), relatively comfortable (6. 1–7. 5), moderate (4. 6–6. 0), inadequate (3. 1–4. 5), tongue stagnation (≤3).
6. *Sweet feeling*: significant (7. 6–9. 0), relatively significant (6. 1–7. 5), still significant (4. 6–6. 0), moderate (3. 1–4. 5), weak (≤3).
7. *Oil content*: rich (++++), more (+++), slightly (++), less (+).

Based on the evaluation results, samples with high oil content were selected for subsequent analysis and designated as H samples, while those with low oil content were labeled as L samples.

### 2.3 Assessment of agronomic traits in tobacco leaves

The lengths, widths, and single - leaf dry weights of tobacco leaves with high oil content and those with low oil content were measured separately. Each measurement was repeated three times, and the average values were calculated. The high - oil and low - oil tobacco leaves were evaluated in terms of the color, oily texture, and softness of dried tobacco leaves, and photographs were taken. The leaf shape index was calculated according to Equation 1 as follows:


(1) Leaf shape index=leaf length/leaf width

### 2.4 Observation microscopic morphology of tobacco leaves

Yunyan 87 dried tobacco leaves with high - oil and low - oil contents were punched and sampled in such a way as to minimize mechanical damage, and the area of the tissue blocks did not exceed 3 mm². The samples were gently rinsed with PBS, and the surface intended for scanning was carefully protected and marked. Subsequently, the samples were rapidly positioned in an electron microscope fixative solution and fixed at room temperature for 2 hours. Then, the samples were transferred to 4°C for storage. The fixed samples were rinsed three times with 0. 1 M PBS (pH 7. 4), each rinse lasting 15 min. An osmium acid solution (1%) was prepared using 0. 1 M PBS (pH 7. 4) and incubated at room temperature in the dark for 1 - 2 hours. Afterward, it was rinsed three times with 0. 1 M PBS (pH 7. 4), each rinse lasting 15 min. The samples were treated with 30, 50, 70, 80, 90, 95, 100, and 100% alcohol for 15 min and finally treated with isoamyl acetate for 15 min. The samples were placed in a critical point dryer for drying. The tobacco leaves were placed close to the conductive carbon film double-sided adhesive and on the sample table of the ion sputtering instrument for gold spraying for approximately 30 s. Scanning electron microscopy (SEM, SU8100, HITACHI, Tokyo, Japan) was utilized to observe and capture images.

### 2.5 Flavor substances detection of tobacco leaves

Based on the sensory evaluation results of the Yunyan 87 oil content carried out by Guizhou China Tobacco Industry Co. , Ltd. , dry tobacco samples with high oil content were selected and labeled H-1, H-2, and H-3, while dry tobacco samples with low oil content were named L-1, L-2, and L-3. Tobacco leaves were mailed to Suzhou Panomik Biomedical Technology Co. , Ltd. (Jiangsu, China) for testing to clarify the characteristic flavor substances of Yunyan 87 tobacco leaves with high-oil content.

#### 2.5.1 Preparation of internal standard solution

An appropriate quantity of the standard (deuterated n - hexanol - d13) was transferred and dissolved in a 50% ethanol aqueous solution. A single - standard mother liquor at a concentration of 10 mg/L was prepared, and the stock solution was refrigerated at 4°C.

#### 2.5.2 Flavor substances extraction

Place the sample in a 15 mL centrifuge tube and dilute the ethanol concentration in the sample to 10% (v/v) with a saturated sodium chloride aqueous solution. The 5 mL of diluted liquor samples was transferred to 20 mL headspace sampling bottles, and 10 µL of the internal standard solution was added to the sample. The transferred sample was incubated for 10 min at 50°C. Before adsorbing the sample, the solid-phase microextraction (SPME) extraction head was aged at 270°C for 10 min. Subsequently, the aged SPME extraction head was transferred to the incubation room, and the sample was adsorbed for 30 min at 50°C. After adsorption, the SPME extraction head was transferred to the gas chromatography (GC) inlet, desorbed at 5 min at 250°C, and then aged for 10 min at 270°C post-injection.

#### 2.5.3 GC×GC analysis

The LECO Pegasus BT 4D (LECO, St. Joseph, MI, USA) GC×GC-TOF MS chromatographic system consists of an Agilent 8890A gas chromatograph (Agilent Technologies, Palo Alto, CA, USA), a dual-stage jet modulator, and a split/splitless injection module. The mass spectrometric system is a high-resolution TOF mass spectrometer. The separation system comprises a first-dimensional chromatographic column, namely Rxi-5Sil MS (30 m × 0. 25 mm × 0. 25 μm, Restek) (Restek, USA), which is suitable for the preliminary separation of volatile and non-polar compounds, and a second-dimensional chromatographic column, namely Rxi-17Sil MS (1. 5 m × 0. 15 mm × 0. 15 μm, Restek) (Restek, USA), which enables secondary orthogonal separation via selective differences. High-purity helium gas (99. 999%) is used as the carrier gas, and an electronic flow controller is employed to maintain a constant flow rate of 1. 0 mL/min, ensuring the reproducibility of retention times. For the first-dimensional chromatographic column Rxi-5Sil MS, the initial temperature is set at 40°C and maintained for 3 min. Subsequently, the temperature is increased at a rate of 5°C/min until it reaches 180°C, after which it is held at this temperature for 1 min. Thereafter, the temperature is further raised at a rate of 4°C/min to 250°C and finally maintained at this final temperature for 3 min. The temperature program of the second-dimensional chromatographic column Rxi-17Sil MS is 5°C higher than that of the first-dimensional chromatographic column. The modulator temperature is always 15°C higher than the column temperature of the second-dimensional chromatographic column, with a modulation period of 8 s, effectively avoiding peak broadening and improving the two-dimensional separation efficiency. The injection system features a high-temperature injection port of 250°C, designed to accommodate various injection modes (pulsed/splitless), enabling effective vaporization of high-boiling-point components (Savareear et al., 2019).

#### 2.5.4 Mass spectrum conditions

The mass spectrometer detector LECO Pegasus BT 4D (LECO, St. Joseph, MI, USA) was employed in this study. The temperature of the mass spectrometric transfer line was set at 250°C, which helps to ensure the stability and accuracy of the analytes during transmission. The ion source temperature was maintained at 250°C, enabling the sample molecules to acquire sufficient energy for efficient ionization. The acquisition rate was 200 spectra/s, allowing for the rapid acquisition of a large amount of mass spectral data while ensuring data integrity. The electron impact ionization energy was set at 70 eV, under which most organic compounds can achieve stable ionization and generate characteristic fragment ions, facilitating the qualitative and quantitative analysis of the samples. The detector voltage was adjusted to 2028 V to ensure accurate detection and amplification of the mass spectral signals, while avoiding signal distortion or increased noise caused by excessively high or low voltages, thereby improving the quality of the mass spectral data. The mass spectrometric scanning range was set from m/z 35 to 550, which can cover the molecular ion peaks and major fragment ion peaks of most organic compounds, showing good adaptability for analyzing various components in complex samples (Tran and Marriott, 2007).

### 2.6 Analysis of flavor substances

Flavor substances primarily comprise volatile flavor components, including hydrocarbons, aldehydes, esters, acids, ketones, alcohols, ethers, phenols and heterocyclic substances. These substances are generated through a series of intricate biochemical reactions during the processing of flavor precursors. Using the PubChem database and classyfire software, the types of detected flavor substances were annotated and analyzed, and the number and relative contents of flavor substances corresponding to each category were analyzed (Li et al., 2023).

#### 2.6.1 Screening of flavor substances

The screening conditions of different substances were as follows: *p* value < 0. 05+VIP > 1 in *t* test or one-way ANOVA test. The experiment adopts aggregated hierarchical clustering, each object was classified into one class, and these classes were merged into larger and larger objects until the end. The data was scaled by pheatmap package, and the hierarchical cluster diagram of relative quantitative values of substances was obtained.

#### 2.6.2 Analysis of sensory flavor characteristics

The flavor of the product comprised the recognizable taste and smell characteristics and a complex of unrecognizable ones. The flavor molecules database (FlavorDB) (https://cosylab.iiitd.edu.in/flavordb) was utilized to analyze and compare the sensory flavor of the substances (Maldonado et al., 2003; Hu et al., 2024). Based on the FlavorDB database, igraph was used to construct the network diagram of sensory flavor.

### 2.7 Statistical analysis

Analysis of variance (ANOVA) was carried out using Statistix 8. 1 (Tallahassee, FL, USA), and the comparison processing method was based on the least significant difference test at the level of 0. 05. Principal component analysis (PCA) was used to downscale and classify the metabolic data to obtain more reliable and intuitive results. All flavor substances in the targeted flavor omics and sensory annotation were obtained from the FlavorDB.

## 3 Results and discussion

### 3.1 Sensory flavor evaluation of tobacco leaves

A sensory evaluation of ten tobacco leaves was carried out, focusing on multiple aspects such as aroma quality, aroma quantity, miscellaneous gas, thrill, sweet feeling, and oil content. The results presented in **Supplementary Table 1**, indicate that sample 1 received the highest total score of 42. 5, while sample 10 obtained the lowest score of 36. 8. Notably, sample 1 of tobacco leaves was characterized by a high oil content, whereas sample 10 exhibited a low oil content. Therefore, sample 1 with sufficient oil content and sample 10 with low oil content were selected for further research and were designated as sample H and sample L, respectively.

### 3.2 Agronomic characteristics of tobacco leaves

**Figure 1:**
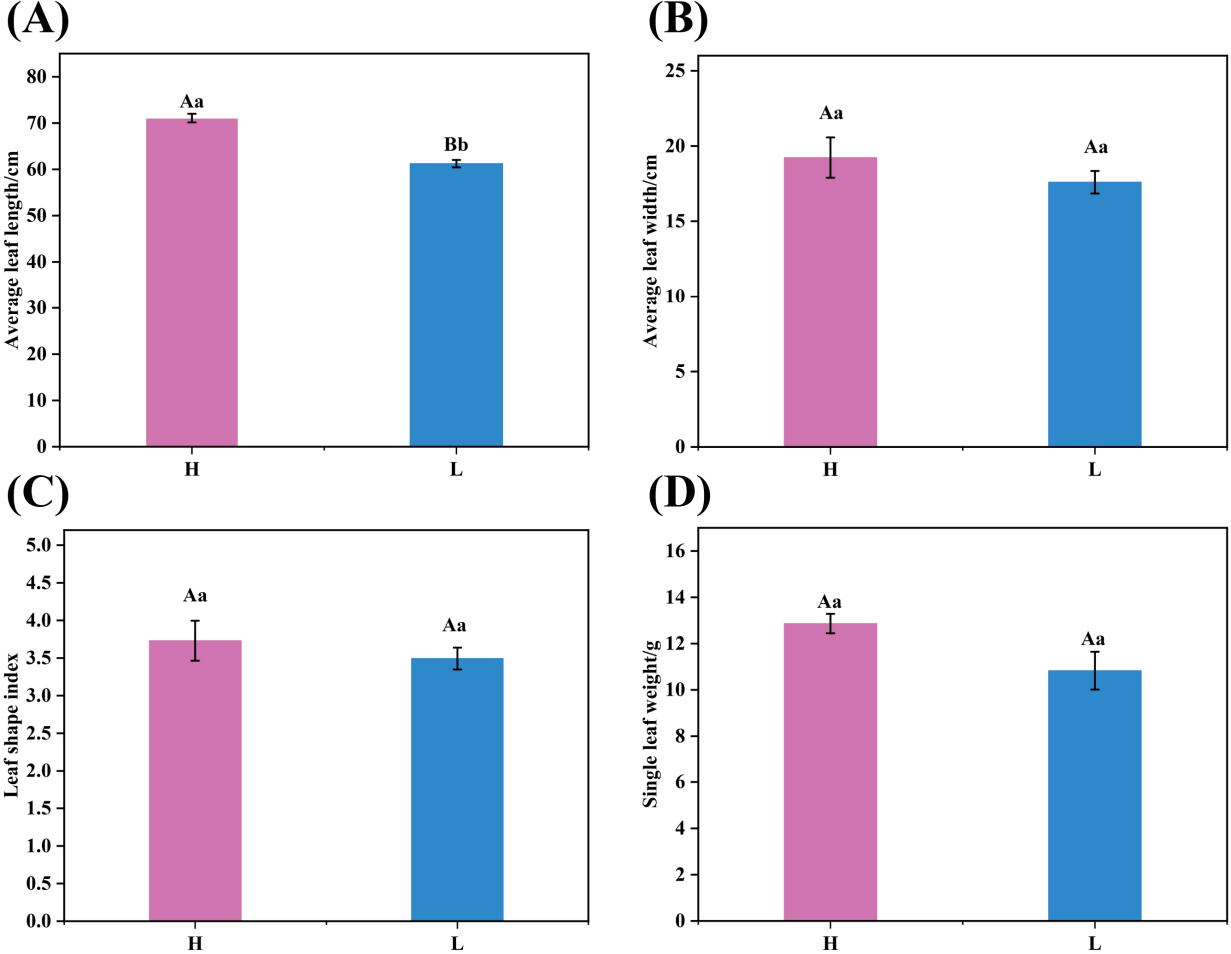
Agronomic characteristics of tobacco leaves. **(A)**Average leaf length, **(B)** average leaf width, **(C)** leaf shape index, and **(D)** single leaf weight. The letters ‘H’ and ‘L’ denote high oil content and low oil content tobacco leaves, respectively. In the bar chart, means sharing the same letter are not significantly different from each other as determined by ANOVA and Duncan’s test (*p* < 0. 05).

The dry average leaf length, average leaf width, leaf shape index, and average single leaf weight of tobacco leaves with different oil content were determined. As illustrated in **Figure 1**, the average leaf length of high-oil tobacco leaves was significantly greater than that of low-oil tobacco leaves, measuring 71. 06 cm and 61. 23 cm, respectively. Although the average leaf width and average single leaf weight of high-oil tobacco leaves were higher than those of low-oil tobacco leaves, the differences were not statistically significant. The leaf shape index shows that tobacco leaves with both levels of oil content were needle - shaped, and this shape was more pronounced in high - oil tobacco leaves.

### 3.3 Appearance morphology of dried tobacco leaves

**Figure 2:**
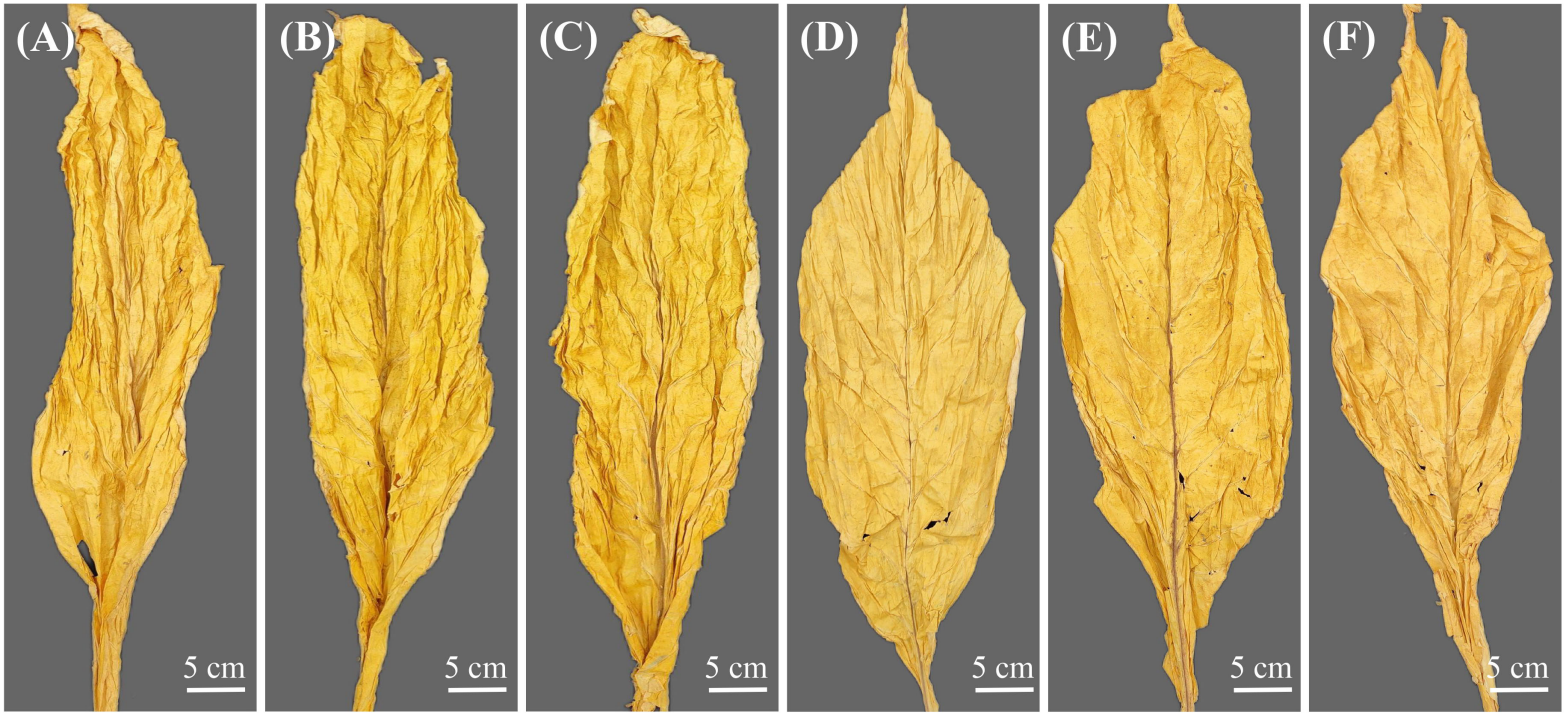
Appearance morphology of dried tobacco leaves with different oils contents. **(A–C)** high-oil tobacco leaves, **(D–F)** low-oil tobacco leaves.

Through the appearance evaluation of dry tobacco leaves with different oil contents, it was found that the front and back of high-oil-content tobacco leaves were generally similar in color. The leaves were of medium thickness, with delicate leaf tissue and obvious suppleness (**Figures 2A–C**). Moreover, the color of the high-oil tobacco leaves was orange and mature, the leaf structure was loose, the oily feeling was strong, and the color fullness and uniformity of the tobacco leaves were excellent. Nevertheless, the low-oil-content tobacco leaves had stiff and thin leaves, poor oiliness, and weak chromaticity (**Figures 2D–F**).

### 3.4 Microscopic morphology of tobacco leaves

**Figure 3:**
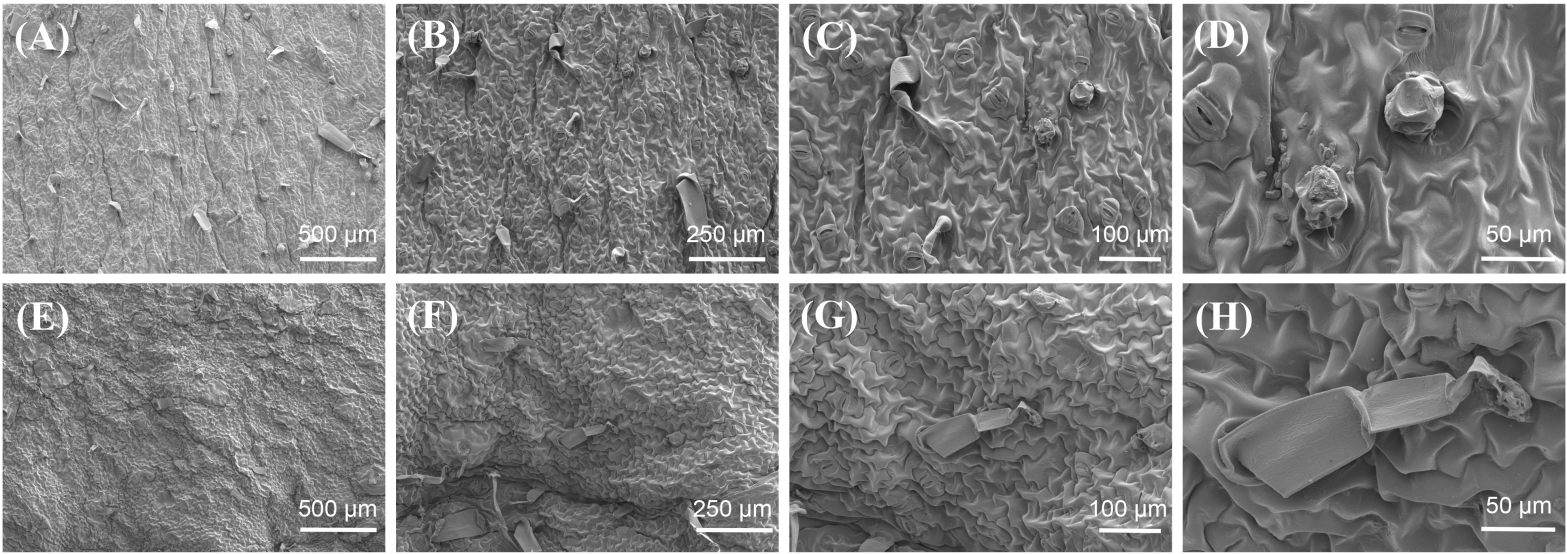
Microscopic morphology of dried tobacco leaves with different oils contents. **(A–D)** high-oil dry tobacco leaves, **(E–H)** low-oil dry tobacco leaves.

The glandular hairs of plants are specialized multicellular structures that originate from epidermal cells and form the first line of defense against abiotic and biotic stresses by forming physical barriers and secreting various substances (Dunn et al., 2011; Djoumbou et al., 2016; Garg et al., 2018). The glandular hairs present on the surface of tobacco leaves possess secretory functions within the epidermal glandular structures of plants. Based on their morphological and structural characteristics, tobacco glandular hairs can be classified into two types: long-stalked glandular hairs and short-stalked glandular hairs. The long-stalked glandular hairs are composed of a multicellular stalk and either a unicellular or multicellular head. In contrast, the short-stalked glandular hairs consist of a unicellular stalk and a multicellular head (Song et al., 2022). The glandular hairs on the surface of tobacco leaves primarily secrete essential oils, resins, waxes, sugars, alcohols, ketones, alkanes, and other substances, which are associated with the aroma and taste of tobacco leaves and the resistance characteristics of tobacco (Wang et al., 2001; Yan et al., 2021). The micro - morphologies of high - oil and low - oil tobacco samples were observed by scanning electron microscopy (SEM). The SEM results of tobacco leaves indicate that the surface glandular hairs of tobacco leaves with high-oil content were richer than those of tobacco leaves with low-oil content (**Figure 3**). Most glandular hairs on the surface of high-oil tobacco leaves were short-stalked glandular hairs, with enlarged glandular heads and rich secretions, cuticle ornamentation was relatively sparse. The results indicated that the oil content of tobacco leaves might be associated with the density and morphology of the glandular hairs on their surfaces.

### 3.5 Total ion flow chromatogram

**Figure 4:**
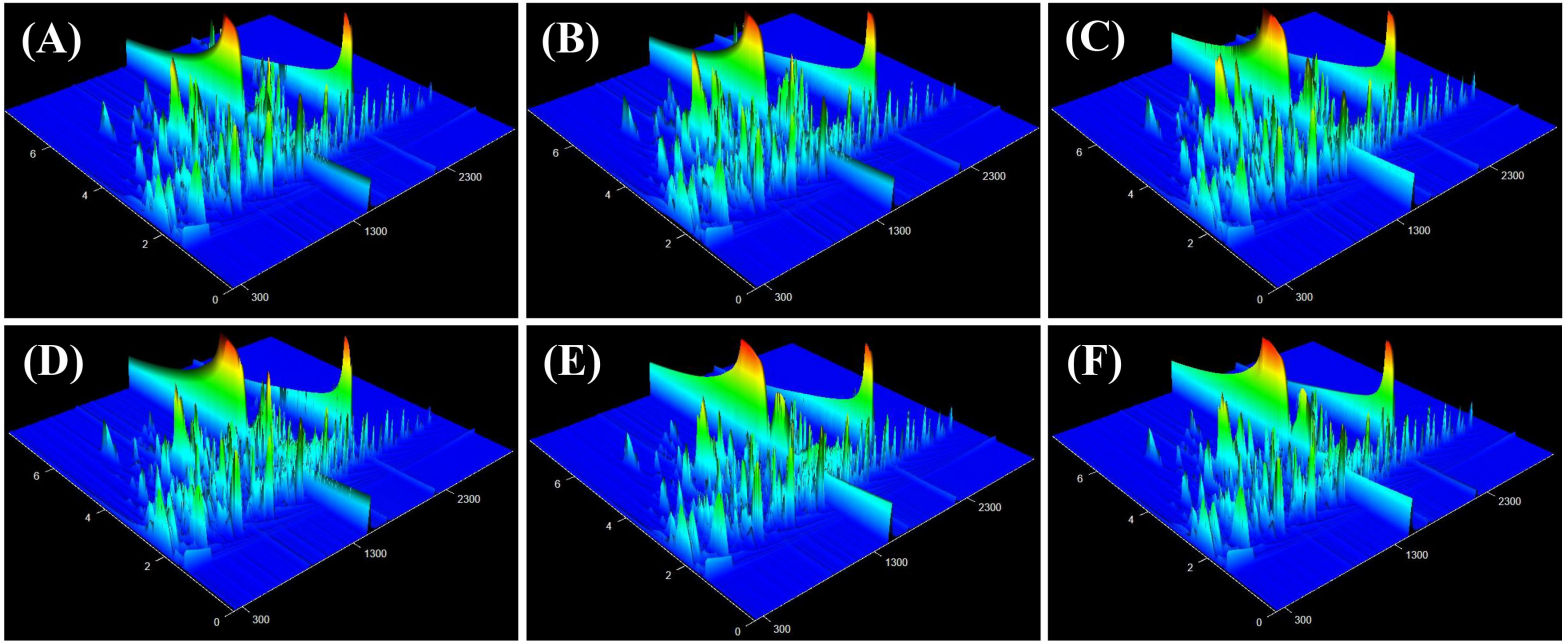
Total ion flow chromatogram of tobacco leaves with different oil contents. **(A–C)** high-oil dry tobacco leaves, **(D–F)** low-oil dry tobacco leaves.

The components isolated through chromatography are subsequently introduced into the mass spectrometer, which performs continuous scanning to gather data. A mass spectrogram was obtained from each scan, and all the ion intensities in each mass spectrogram were added to obtain the total ion current intensity. In this study, GC×GC-TOF MS was employed to analyze the volatile components of tobacco samples with high and low oil contents. The abscissa and ordinate represent the one-dimensional and two-dimensional retention times (in seconds), respectively (**Figure 4**). The color and peak height reflect the intensity of the ion response, with a redder color indicating a higher response strength. The overall peak occurrence rate in the three-dimensional total ion current chromatogram of each sample was satisfactory, suggesting that the samples contained a large number of volatile substances.

### 3.6 Principal component analysis

**Figure 5:**
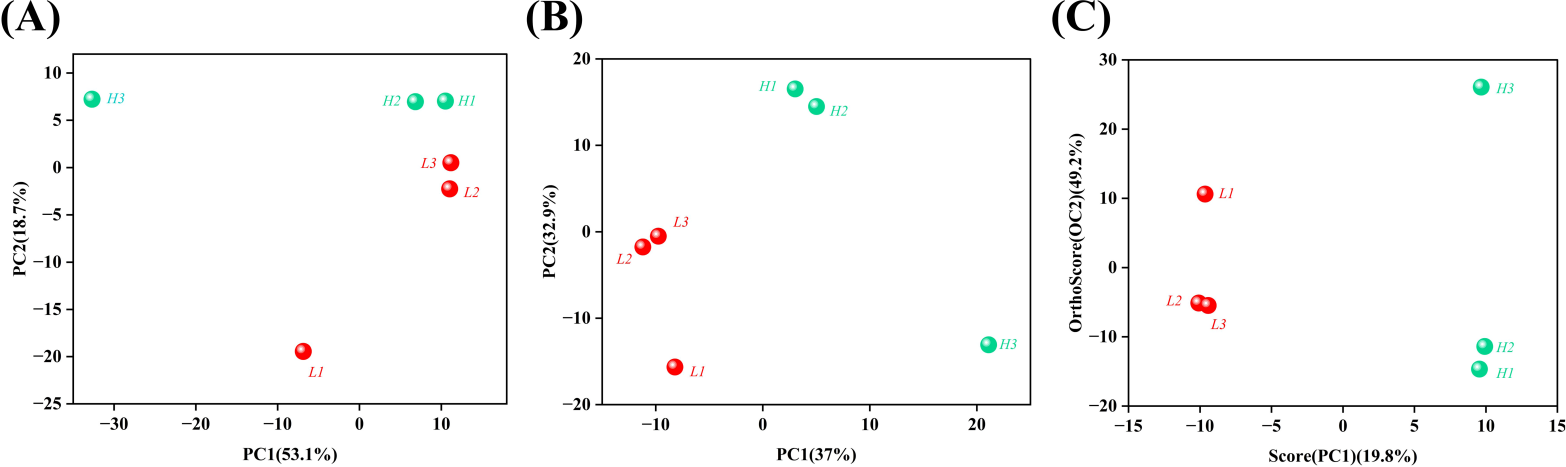
Principal component analysis (PCA) of tobacco leaves with different oil contents. **(A)** PCA Score Plot, **(B)** PLS-DA Score Plot, **(C)** OPLS-DA Score Plot. The letters ‘H’ and ‘L’ denote high oil content and low oil content tobacco leaves, respectively.

The principal component analysis (PCA) model reflects the original state of the metabolomic data, facilitating an understanding of the overall data situation. In particular, it is helpful for identifying and eliminating abnormal samples and enhancing the accuracy of the model. The score of each sample for each principal component represents its spatial coordinates within the calculated mathematical model, intuitively reflecting the distribution of each sample in the model space. The degrees of aggregation and dispersion of the samples can be visualized in the PCA score chart. The closer the distribution points of the samples, the closer the composition and concentration of the variables/molecules contained in these samples. As illustrated in **Figure 5**, each point was segregated into two clusters. The tobacco samples with low and high oil contents were positioned on the left and right sides of PC1, respectively. PCA revealed that there was a significant difference in the flavor substances between tobacco leaves with high oil content and those with low oil content.

### 3.7 Statistical analysis of identification flavor substances

**Figure 6:**
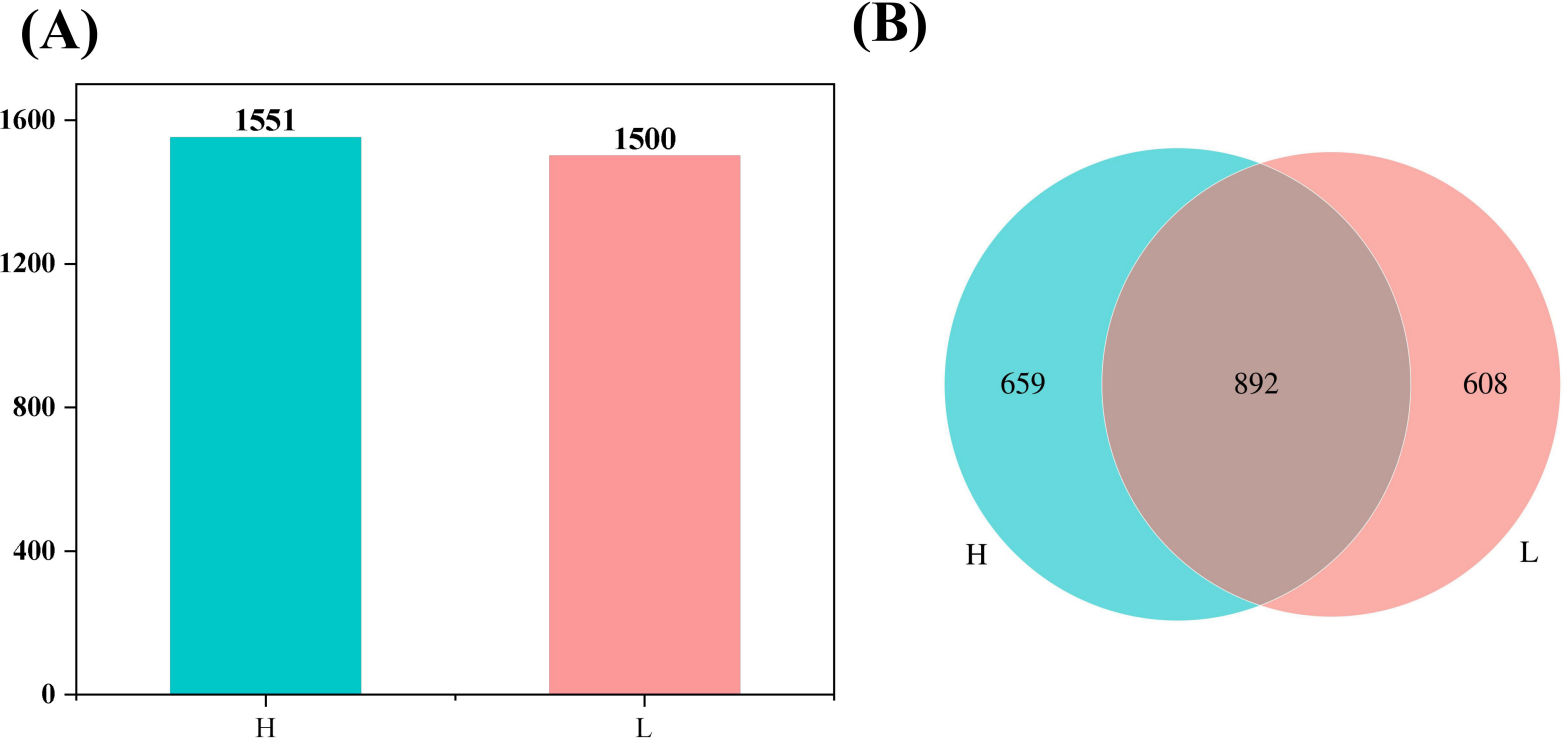
Statistical analysis of identification flavor substances of tobacco leaves with different oil contents. **(A)** Column chart of the number of identification substances; **(B)** Venn diagram of the number of identification substances. The letters ‘H’ and ‘L’ denote high oil content and low oil content tobacco leaves, respectively.

The tobacco leaves with different oil contents were analyzed, revealing the detection of 1551 flavor substances in high-oil tobacco and 1500 flavor substances in low-oil tobacco. Within the detectable range, the flavor substances detected in high-oil tobacco leaves were found to be more plentiful than those in low-oil tobacco leaves. As illustrated in **Figure 6**, a total of 892 types of flavor substances were identified in both high-oil and low-oil tobacco. Among them, 659 types of specific flavor substances were detected in high-oil tobacco. This indicates that the specific flavor substances in high-oil tobacco samples were more numerous than those in low-oil tobacco samples.

### 3.8 Analysis of flavor substances of tobacco leaves

**Figure 7:**
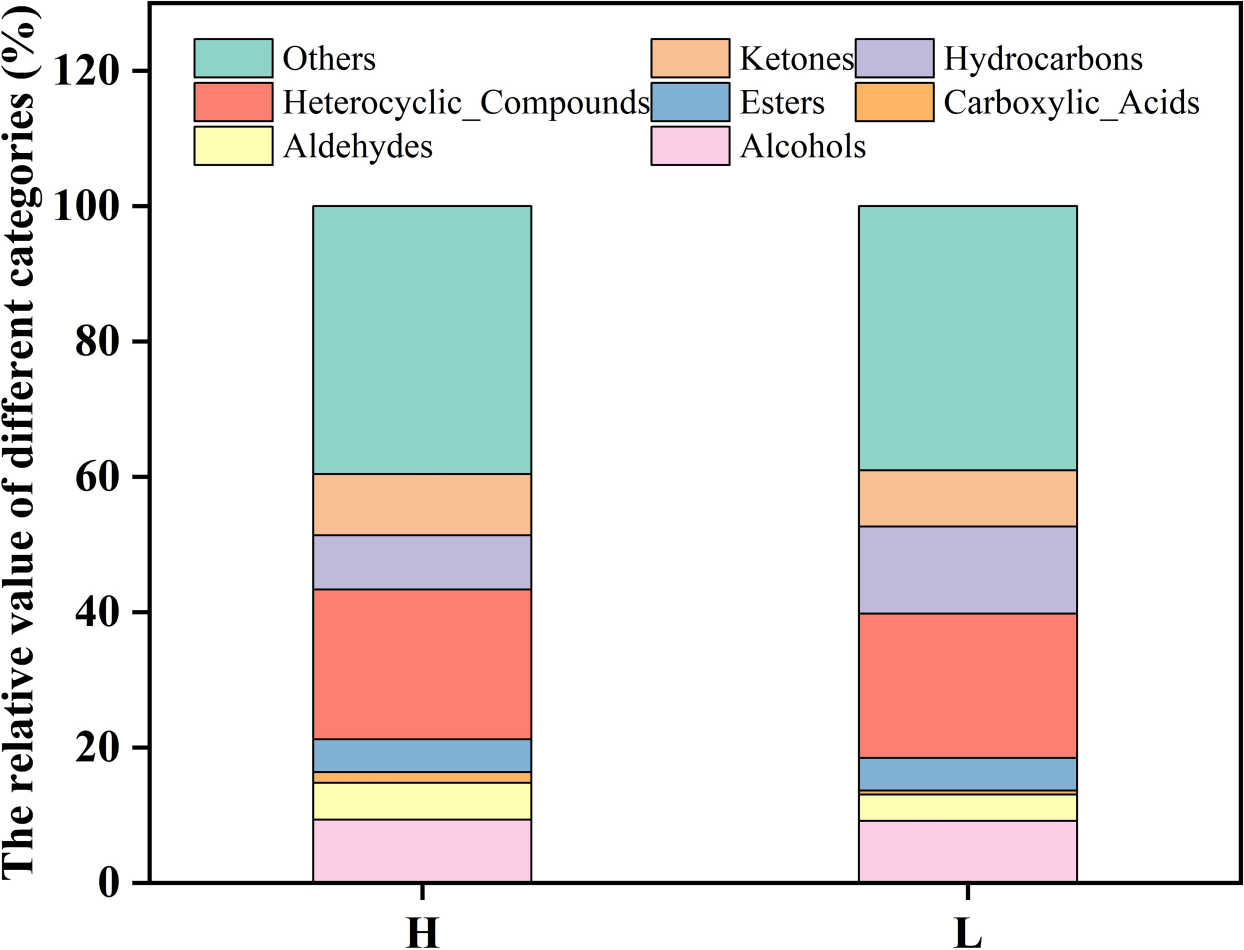
Analysis of flavor substances in tobacco leaves with different oils. The letters ‘H’ and ‘L’ denote high oil content and low oil content tobacco leaves, respectively.

Flavor results from the interaction among taste, pre-nasal olfactory perception, and post-nasal olfactory perception and is frequently termed taste and aroma (Meyberg et al., 1991). Flavor substances consist of esters, acids, hydrocarbons, aldehydes, ketones, alcohols, ethers, phenols, and heterocyclic substances, which are produced via a series of complex biochemical reactions during the processing of flavor precursors (Gang et al., 2001). The PubChem database and classyfire software were employed to analyze the differences in the relative content of flavor substances between high-oil and low-oil tobacco leaves. As shown in **Figure 7**, the proportions of alcohols (9. 33%), aldehydes (5. 45%), ketones (9. 01%), esters (4. 86%), carboxylic acids (1. 58%), heterocyclic substances (22. 13%) and other volatile substances (39. 61%) in high-oil tobacco leaves were higher than those in low-oil tobacco leaves. The relative content of hydrocarbons in high-oil tobacco leaves (8. 02%) was found to be lower than that in low-oil tobacco leaves (12. 88%). Neutral aromatic substances in tobacco, including alcohols, ketones, and esters, play a crucial role as flavor substances in enhancing the quality of tobacco products (Kroumova and Wagner, 2003; Goff and Klee, 2006). The main esters identified in tobacco leaves were ethyl octanoate, ethyl oleate, ethyl laurate, hexadecanoic acid methyl ester, and nonanoic acid methyl ester. The majority of esters in tobacco are higher fatty acid esters, which can serve as carriers of flavor substances and soften the smoke.

### 3.9 Screening of different flavor substances

**Figure 8:**
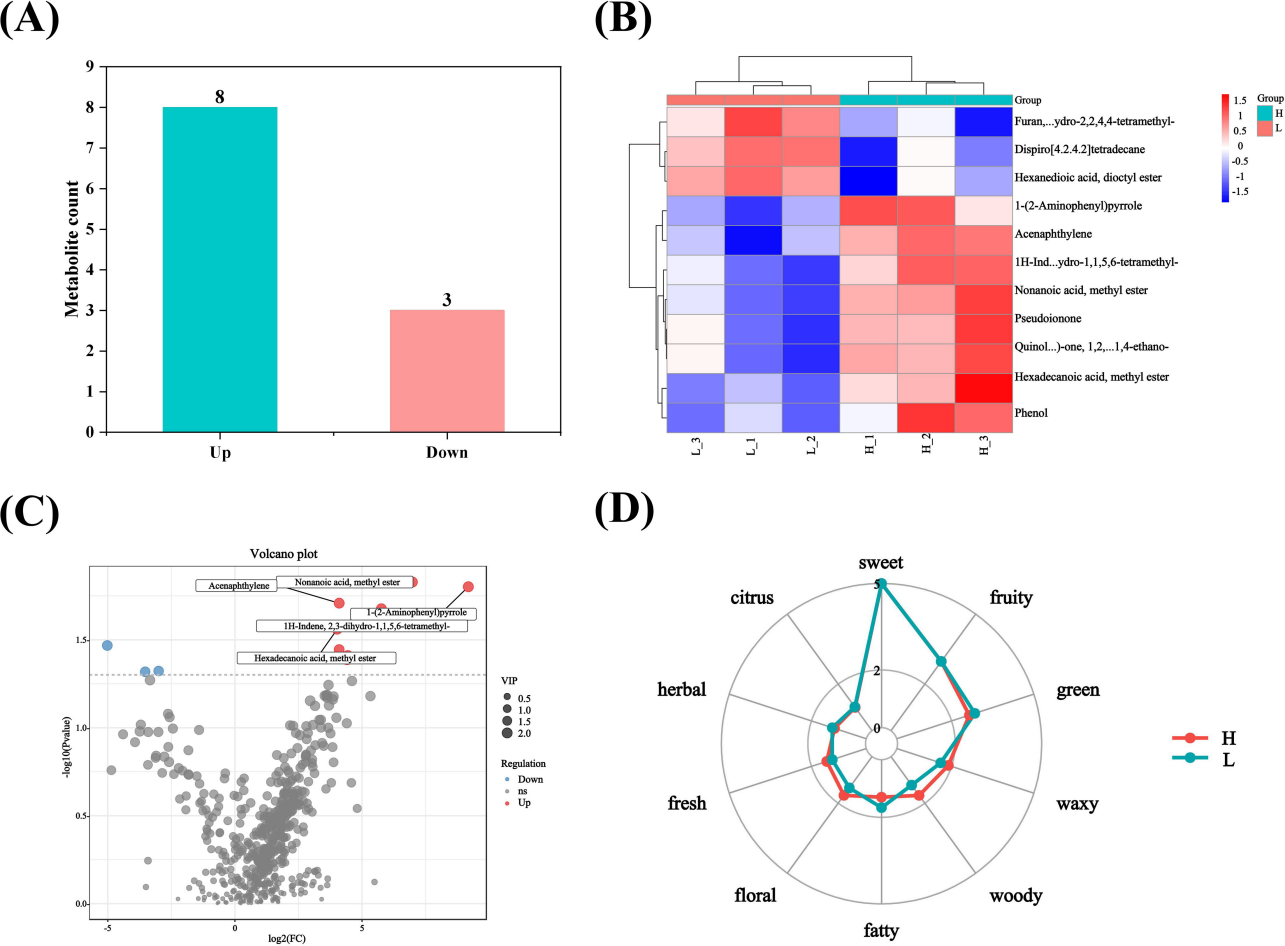
Screening of various substances in tobacco leaves and analysis of their sensory flavor characteristics. **(A)** Statistical column chart of different flavor substances; **(B)** heat map of different flavor substances; **(C)** volcano plot of different flavor substances; **(D)** sensory flavor characteristic chart of tobacco leaves with different oil contents. The letters ‘H’ and ‘L’ denote high oil content and low oil content tobacco leaves, respectively.

After screening, different flavor substances were identified in the list of high-oil and low-oil tobacco leaves. The optional screening conditions for the related substances were *p*-value <0. 05 + VIP >1 in the *t*-test or one-way ANOVA. As shown in **Figure 8**, the comparison of low-oil and high-oil tobacco leaves were presented. The screening outcomes for various flavor substances revealed that there were 573 distinct metabolites in high-oil and low-oil tobacco leaves. Among them, eight metabolites were significantly up-regulated, while three were significantly down-regulated. The significantly down-regulated flavor substances included dispiro [4. 2. 4. 2] tetradecane; furan, tetrahydro-2,2,4,4-tetramethyl; hexanedioic acid, dioctyl ester. The flavor substances that were up-regulated included 1-(2-Aminophenyl) pyrrole; 1*H*-indene, 2,3-dihydro-1,1,5,6-tetramethyl; pseudoionone; acenaphthylene; hexadecanoic acid methyl ester; nonanoic acid methyl ester; phenol; quinolin-6(7 *H*)-one, 1,2,3,4,8,8a-hexahydro-1,4-ethano. The oil-related substance hexadecanoic acid methyl ester and aroma-related substances nonanoic acid methyl ester and pseudoionone were significantly higher in the high-oil tobacco than in the low-oil tobacco. Therefore, the content of hexadecanoic acid methyl ester can be used as an important indicator for evaluating the oil content of tobacco leaves, and nonanoic acid methyl ester and pseudoionone may be important substances influencing the aroma of tobacco leaves.

### 3.10 Analysis of sensory flavor characteristics

**Figure 9:**
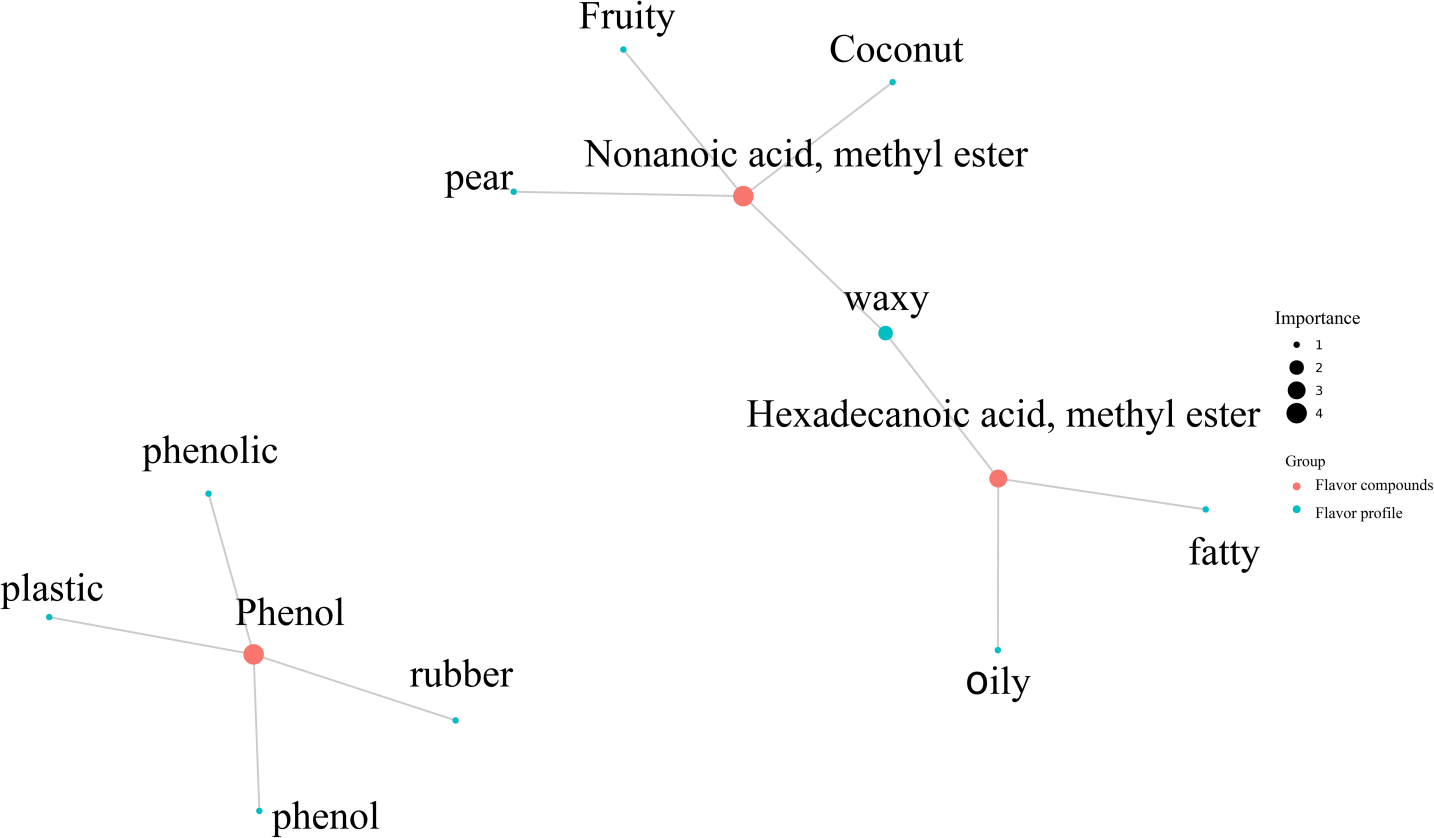
Correlation network diagram of sensory flavor characteristics and flavor substances.

The differences in sensory flavor characteristics between high-oil and low-oil tobacco leaves was compared using the FlavorDB. Ten attributes were used to describe the sensory flavor of tobacco leaves, namely: sweet, fruity, green, waxy, fatty, woody, fresh, citrus, herbal, and floral. As depicted in **Figure 8D**, the sensory flavors of fresh, floral, woody, and waxy notes in high-oil tobacco leaves were more pronounced than those in low-oil tobacco leaves. The analysis of sensory flavor characteristics indicated that the predominant flavors of high - oil and low - oil tobacco samples were honey and sweetness. Based on the FlavorDB, igraph was employed to construct a network diagram depicting the relationship between the flavor substances and sensory characteristics (**Figure 9**). The green circles denote sensory characteristics, while the red circles represent flavor substances. The size of the green circles is proportional to the number of flavor substances associated with the sensory characteristics, with larger circles indicating greater importance of the sensory characteristics. The larger the red circle, the greater the number of sensory characteristics associated with the flavor compound, and the more significant the role of the flavor substance. For example, the flavor substance hexadecenoic acid methyl ester influences the oil, fat, and fatty content of tobacco leaves. Meanwhile, nonanoic acid methyl ester, which has a coconut aroma, enhances the fruit aroma of tobacco leaves.

## 4 Conclusion

The oil content of tobacco leaves serves as a crucial index for quality assessment. Tobacco leaves with higher oil content have sufficient aroma and superior aroma quality. This study aims to explore the microstructure and distinctive flavor substances of Yunyan 87 high-oil-content tobacco leaves. We analyzed Yunyan 87 tobacco leaves with different oil levels using SEM and GC×GC-TOF MS. It was found that the glandular hairs on the surface of Yunyan 87 leaves with high oil content were more abundant than those on the surface of Yunyan 87 leaves with low oil content. The majority of glandular hairs on the surface of high-oil tobacco leaves were characterized by short stalks and enlarged glandular heads, with rich secretions and relatively sparse cuticle ornamentation. Overall, a total of 1551 flavor substances were detected in the high-oil tobacco samples, whereas 1500 flavor substances were identified in the low-oil tobacco samples, suggesting that the high-oil tobacco contains a greater abundance of small molecular weight metabolites. Among these flavor substances, eight were found to be up-regulated, while three were down-regulated. Notably, compared with those in low - oil tobacco samples, the levels of oil - related substances (such as hexadecanoic acid methyl ester) and aroma - related substances (including nonanoic acid methyl ester and pseudoionone) were significantly higher in high - oil tobacco samples. The content of hexadecanoic acid methyl ester could potentially serve as a crucial indicator for assessing the oil content of tobacco leaves. Meanwhile, nonanoic acid methyl ester and pseudoionone may play significant roles in modulating the aroma of these leaves. This research offers a theoretical foundation for future investigations into the regulatory mechanisms and synthesis pathways related to the aroma-generating traits of tobacco.

## Data Availability

The original contributions presented in the study are included in the article/**Supplementary Material.** Further inquiries can be directed to the corresponding author.
