# Supplementary material for: Microstructure observation and flavor substances excavation of Yunyan 87 tobacco leaves with different oil contents
Source: Front Plant Sci. 2025 Mar 5;16:1537924. doi: 10.3389/fpls.2025.1537924 (PMC11920166; doi:10.3389/fpls.2025.1537924)
Supplement: Supplementary file 1 [file Table1.doc]

| **Table Supplementary 1.** Sensory evaluation of tobacco leaves with different oil content | | | | | | | | | | |
| --- | --- | --- | --- | --- | --- | --- | --- | --- | --- | --- |
| Evaluating indicator | Sample1 | Sample  2 | Sample  3 | Sample  4 | Sample  5 | Sample  6 | Sample  7 | Sample  8 | Sample  9 | Sample  10 |
| Oil content | ++++ | ++++ | ++++ | +++ | +++ | ++ | ++ | ++ | ++ | + |
| Aroma quality | 7.4 | 7.3 | 7.1 | 7.1 | 7 | 7 | 7 | 7 | 7 | 6.4 |
| Aroma quantity | 7.1 | 7.3 | 7.5 | 7.3 | 7.1 | 7 | 7 | 7 | 6.5 | 6.4 |
| Miscellaneous gas | 7 | 7 | 7 | 7 | 7 | 6.8 | 6.5 | 6.7 | 6.7 | 6 |
| Thrill | 7 | 7 | 7 | 7 | 6.7 | 6.8 | 7 | 6.5 | 7 | 6.1 |
| Agreeable aftertaste | 7 | 7 | 7 | 6.9 | 6.7 | 6 | 6.6 | 6.6 | 6.5 | 6.1 |
| Sweet feeling | 7 | 6.8 | 6.8 | 6.5 | 6.8 | 6.6 | 6 | 6.3 | 6.1 | 5.8 |
| Total score | 42.5 | 42.4 | 42.4 | 41.8 | 41.3 | 40.2 | 40.1 | 40.1 | 39.8 | 36.8 |
